# Crosstalk Between Cortical Plasticity and Peripheral Nervous Injury: Insights, Mechanisms, and Future Directions

**DOI:** 10.1002/cns.71090

**Published:** 2026-09-01

**Authors:** Cong Cheng, Dong Gao, Yaojie Shen, Xi Wang, Junxi Dai, Yaoxian Xiang, Zongyuan Jiang, Zongqi You, Junjian Jiang

**Affiliations:** ^1^ Department of Hand Surgery People's Hospital of Longhua Shenzhen China; ^2^ Shanghai Key Laboratory of Forensic Medicine Institute of Forensic Science, Ministry of Justice Shanghai China; ^3^ Clinical Experimental Centre Zhejiang Hospital of Integrated Traditional Chinese and Western Medicine Hangzhou Zhejiang China; ^4^ Department of Anesthesiology, Sir Run Run Shaw Hospital, School of Medicine Zhejiang University Hangzhou China; ^5^ Department of Hand Surgery Huashan Hospital, Fudan University Shanghai China; ^6^ Department of Neurosurgery Jinling Hospital, Nanjing University Nanjing China

**Keywords:** cortical plasticity, epidemiology, future directions, mechanisms, peripheral nervous injury

## Abstract

**Introduction:**

Cortical plasticity is essential for functional recovery after peripheral nervous injury (PNI). Different injury types, from compressive to metabolic neuropathies, lead to distinct patterns of cortical reorganization, calling for a better understanding of the mechanisms and treatment options for each injury type.

**Methods:**

This review summarizes current evidence from preclinical, neuroimaging, electrophysiological, and clinical studies on the link between cortical plasticity and PNI.

**Results:**

The review describes the key molecular pathways that shape adaptive and maladaptive cortical reorganization across injury types, examines the value of neuroimaging and blood‐based biomarkers for tracking plasticity, and evaluates the current evidence for drug and rehabilitation treatments. Major gaps remain, including the limited testing of biomarkers across different injury types, the lack of PNI‐specific clinical trials, and the difficulty of applying laboratory findings to personalized treatment.

**Conclusions:**

Moving forward, progress depends on validating biomarkers for each injury type, conducting well‐designed trials in PNI patients, and combining drug, rehabilitation, and whole‐body treatment approaches. This review offers a practical guide for turning mechanistic knowledge into clinical focus in PNI‐related cortical plasticity.

## Graphical abstract

Peripheral nerve injury induces dynamic cortical plasticity, driven by molecular mechanisms such as BDNF and neurotransmitter systems. This review synthesizes subtype‐specific plasticity patterns: compressive injuries trigger rapid reorganization, while metabolic neuropathies lead to maladaptive changes. We highlight emerging diagnostic tools, therapeutic strategies, and future directions for personalized neurorehabilitation.

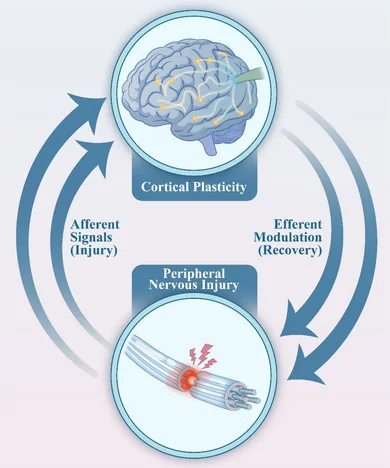

## 1 Introduction to Cortical Plasticity in Peripheral Nervous Injury

### 1.1 Definition and Mechanisms of Cortical Plasticity

**FIGURE 1:**
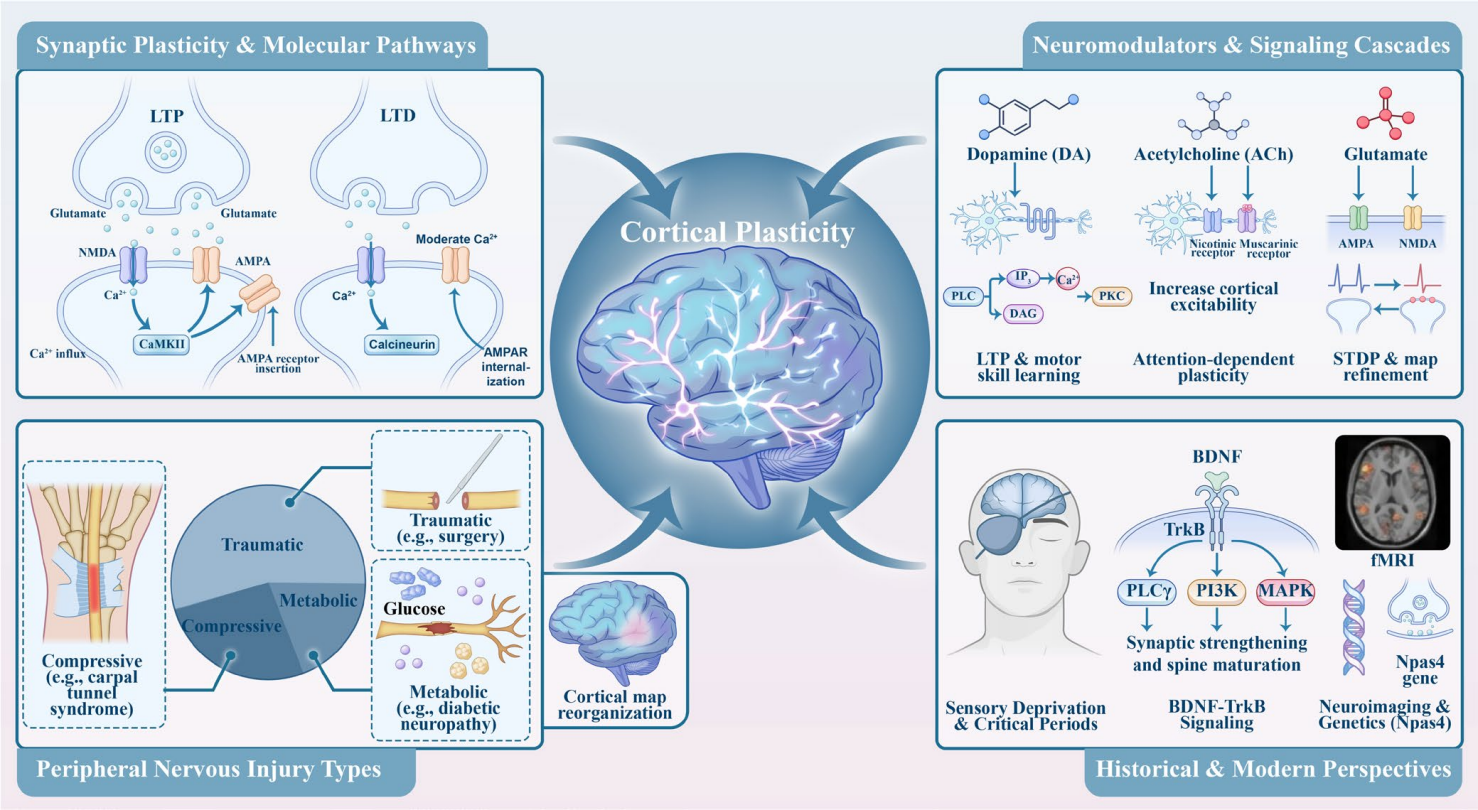
Definition and mechanisms of cortical plasticity.

The brain is always capable of adapting to experience, learning, or injury. This occurs due to the inexhaustible adaptability of its neural circuits—cortical plasticity [1, 2]. This property is fundamentally an ability of the nervous system to adjust itself to changes in the environment. The ways that the brain can change itself are complicated but critical to understanding (Figure 1).

Synaptic plasticity is the most fundamental source of neuronal adaptability. Long‐term potentiation (LTP) and long‐term depression (LTD) are driven by well‐defined molecular cascades. High frequency stimulation activates NMDA receptors, leading to Ca^2+^ influx and subsequent CaMKII/MAPK/CREB signaling, which promotes AMPA receptor insertion and the expression of LTP [3, 4]. Conversely, low‐frequency stimulation triggers moderate NMDA‐dependent Ca^2+^ entry, activating calcineurin and PP1, and ultimately inducing AMPA receptor internalization during LTD [5]. LTP is considered a cellular substrate for learning and memory, and it can be induced in the motor cortex through LTP‐like plasticity via paired associative stimulation. The impact of Parkinson's disease on the motor cortex has been researched using short‐term plasticity and LTP as a framework. Thus, in a study of Parkinson's disease patients, subthalamic nucleus deep brain stimulation (DBS) with dopaminergic medications has been shown to restore LTP‐like plasticity in the motor cortex [6]. The therapeutic action of the treatment may be ascribed to this restoration.

Multiple factors contribute to cortical plasticity. Brain‐derived neurotrophic factor (BDNF) is a neurotrophic protein that plays important roles in cognitive function during this process. BDNF enhances synaptic plasticity through TrkB‐mediated activation of PLCγ, PI3K, and MAPK pathways, which promote synaptic strengthening and dendritic spine maturation [7, 8]. In addition, BDNF is necessary for the survival, differentiation and synaptic plasticity of neurons. Patients with psoriasis have low BDNF plasma concentrations. Since psoriasis is a chronic systemic inflammatory disease, BDNF may be linked to cortical plasticity in stress disorders [9]. Neurotransmitters like dopamine (DA), acetylcholine (ACh), and glutamate also modulate cortical plasticity. DA modulates cortical plasticity primarily through D1‐receptor–dependent activation of the phospholipase C (PLC) pathway, leading to the production of IP_3_ and DAG and subsequent PKC activation. This cascade enhances intracellular Ca^2+^ signaling and supports LTP‐like plasticity during motor skill learning [10, 11, 12]. In addition to DA, ACh contributes to cortical plasticity by increasing cortical excitability through nicotinic and muscarinic receptors, thereby facilitating attention‐dependent learning [13, 14]. Glutamate, acting via AMPA and NMDA receptors, is essential for spike‐timing‐dependent plasticity (STDP), a form of Hebbian learning strongly involved in sensory and motor map refinement [15].

### 1.2 Overview of Peripheral Nervous Injury

PNI can be classified into several types, each with its own characteristics and implications. Traumatic nerve injuries are common, often resulting from accidents, such as motor vehicle collisions or falls. For example, obturator nerve injury can occur during laparoscopic lymphadenectomy. In a reported case, a 29‐year‐old woman had a complete dissection of the obturator nerve during the procedure, which was immediately repaired via a laparoscopic approach [16]. Such iatrogenic nerve injuries are common types of nerve injuries. Medical causes, such as central venous catheterization, are generally responsible for the injuries, and these may also lead to mechanical complications, including nerve injury. While these injuries are less frequent, they can become life‐threatening if not treated [17]. Another common form of PNI is chemotherapy‐induced peripheral neurotoxicity (CIPN). Drugs such as vincristine, thalidomide, and eribulin used in cancer treatment cause CIPN. This condition is generally characterized as a mild to moderate axonal, length‐dependent sensory polyneuropathy [18].

Compressive neuropathies result from nerve compression, which can range from a simple anatomical abnormality to a tumor or cyst. Carpal tunnel syndrome (CTS) is a representative example, occurring when the median nerve is compressed in the wrist. Moreover, chronic illnesses such as diabetes mellitus may cause diabetic peripheral neuropathy (DPN). DPN is the most common form of neuropathy. A comparison between Bangladeshi migrants and people born in Greece with diabetes found polyneuropathy was present in 48% and 58% of participants respectively, and the assessment also noted between‐group differences with regard to large versus small fiber neuropathies [19]. Both ethnic and genetic factors play a role in the development of diabetic neuropathy.

Collectively, peripheral nerve injury alters afferent sensory input to the cortex, leading to cortical map reorganization, which can be adaptive or maladaptive depending on the extent and chronicity of the injury [20, 21].

### 1.3 Historical Perspectives on Cortical Plasticity Research

For several years, cortical plasticity has been studied, evolving with advances in techniques and understanding. Initially, it was believed that the brain was a static structure with minimal capacity for modification. Yet, as research progressed, compelling evidence of the brain's ability to adapt and reorganize began to surface.

Throughout the years, sensory deprivation research has substantially increased our understanding of neural development and plasticity. Research on congenital cataracts shows that untreated cases during early development cause permanent visual acuity deficits known as amblyopia. This demonstrates that sensory experience is important for establishing neural circuits and supporting their maturation into adulthood. Later studies have shown that enriched environmental protocols in adults enhance cortical plasticity, improving amblyopia recovery and indicating that the brain remains plastic beyond classical critical periods [22].

Recent advancements in neuroimaging technologies, notably functional magnetic resonance imaging (fMRI) and electroencephalography (EEG), have revolutionized our understanding of cortical plasticity [23]. Using these innovative methods, researchers have been able to closely monitor changes in brain activity and connections, which has advanced the understanding of the mechanisms of plasticity. According to fMRI studies, motor learning is related to large‐scale changes in resting‐state functional connectivity.

Furthermore, our understanding of such mechanisms has been expanded by the discovery of molecular mediators of cortical plasticity such as BDNF. The identification of principal genes and related proteins, which mediate synaptic plasticity, has opened new research opportunities. One example of this is the exploration of Npas4, which is implicated in barrel cortex inhibitory control, and its role in the molecular process by which cortical plasticity occurs [24]. It promotes the formation of inhibitory synapses and regulates excitatory‐inhibitory (E/I) balance in cortical circuits, thereby shaping experience‐dependent plasticity [25, 26].

## 2 Epidemiology of Peripheral Nervous Injury and Cortical Plasticity

### 2.1 Incidence and Prevalence of Peripheral Nervous Injury

The types of injury and the population surveyed influence the incidence and prevalence of PNI. Traumatic peripheral nerve injuries are relatively common, especially among younger populations. In one study of 1998 patients undergoing hip arthroplasty, although there was a high overall rate of adverse events, only 8% of patients made claims for major preventable adverse events related to a nerve injury [27].

DPN is one of the major complications associated with diabetes mellitus, especially in Type 2 diabetes. In a longitudinal cohort study of 477 participants with Type 2 diabetes, 28% had DPN at baseline and 20% developed new or progressive neuropathy over a median of 6.2 years of follow‐up [28]. Correspondingly, the incidence of chemotherapy‐induced peripheral neurotoxicity largely depends on the chemotherapeutic agent used. Regarding HIV‐associated neuropathy in antiretroviral therapy, a study conducted among untreated symptomatic HIV‐infected individuals from Uganda and Zimbabwe starting zidovudine‐containing antiretroviral therapy found that 12% of the participants developed a new episode of PNI, which had an incidence of 2.12 per 100 person‐years [29]. These findings underscore the multifactorial etiology and clinical heterogeneity of peripheral neuropathies across various patient populations.

Neuropathic pain, frequently linked to peripheral nerve injury, represents a considerable public health concern. In a national multicenter trial, 11% of individuals with spinal cord injury reported neuropathic pain, which showed a significant and independent association with cardiovascular disease [30].

### 2.2 Demographic Factors Influencing Cortical Plasticity

Demographic factors exert a significant influence on cortical plasticity, with age being one of the most prominent determinants. In pediatric populations, the brain exhibits heightened plasticity, facilitating superior recovery from neurological insults. A study examining children with traumatic brain injury revealed that individuals sustaining injury at an earlier age experienced reduced quality of social relationships in adulthood, underscoring the vulnerability of younger brains to the long‐term consequences of injury on social functioning [31].

In contrast, cortical plasticity diminishes in older adults, which may contribute to compromised cognitive recovery. In patients with late‐onset multiple sclerosis (LOMS), cognitive performance was found to be inferior to that in adult‐onset multiple sclerosis (AOMS) after adjusting for age and disease duration [32]. A later age of MS onset was associated with worse cognitive functioning, likely attributed to the decline in neuroplasticity within an aging neurophysiological system.

Gender contributes significantly to cortical plasticity as well. Current evidence reveals that females respond differently to stimuli that induce changes in cortical plasticity. Studies on early‐life adversities and chronic pain show that females experience more pain symptoms and males have higher neuroimmune responses [33]. In addition, genetic characteristics can alter demographic factors' effect on plasticity. Specifically, the BDNF Val66Met polymorphism affects recovery in stroke patients [34]. Those who are carriers of the Met allele rely more heavily on subcortical plasticity than how much most people use it. By contrast, the ValVal genotype has enhanced intracortical plasticity.

### 2.3 Subtype‐Specific Epidemiology and Implications for Cortical Plasticity

The epidemiological profiles of peripheral nerve injury subtypes profoundly influence their modulation of cortical plasticity, highlighting the need for subtype‐tailored therapeutic strategies. Compressive injuries, such as CTS—the most common entrapment neuropathy with a prevalence of approximately 5% in specific at‐risk female cohorts—often present acutely or subacutely due to repetitive mechanical stress or trauma [35]. This rapid deafferentation triggers accelerated cortical remodeling, with longitudinal functional magnetic resonance imaging (fMRI) studies demonstrating synaptic reorganization in the primary somatosensory cortex (S1) within as little as 8 weeks post‐onset, including unmasking of latent synapses and expansion of adjacent cortical representations [36].

In contrast, metabolic injuries such as DPN and CIPN exhibit a chronic, insidious progression driven by hyperglycemia‐induced oxidative stress or neurotoxic agents, leading to gradual axonal degeneration, delayed cortical plasticity (typically > 6 months in longitudinal DM cohorts), and maladaptive sensitization [37]. These differences underscore how compressive injuries prioritize acute input loss, fostering rapid adaptive plasticity via heightened excitatory‐inhibitory balance shifts, whereas metabolic subtypes amplify chronic inflammatory cascades, increasing the risk of persistent central pain amplification. The 2024 cohort data suggest a potentially stronger association between S1 activation reduction and shorter symptom duration in CTS patients compared to DPN cohorts, emphasizing the urgency of early subtype‐specific interventions to harness plasticity windows [36, 38]. This epidemiological heterogeneity not only informs prevalence modeling but also guides prognostic tools for plasticity‐driven recovery trajectories.

### 2.4 Global Trends in Peripheral Nervous Injury Research

Recently, there has been an increase in global interest in PNI. According to a bibliometric analysis, there has been a rise in publications addressing the surgical management of upper limb peripheral nerve injuries [39]. Notably, the US and China are both major contributors to these investigations.

Research has increasingly addressed various areas in the nerve injury and regeneration field. New diagnostic techniques have been developed to enhance the ability to diagnose and assess nerve injury and regeneration using imaging. Advances in medical imaging have also contributed to better management of peripheral nerve injury [40]. Nevertheless, to improve the management of PNI, noninvasive medical imaging techniques are being optimized for decision‐making. One important research area involves discovering innovative treatments. The tissue engineering technology that incorporates seed cells, neurotrophic factors and scaffold material holds promise for application in long‐gap and extensive nerve injury in patients [41].

The last few years have seen an increasing interest in understanding the molecular and cellular mechanisms involved in the injury and regeneration of peripheral nerves. Current research has focused on how Schwann cells, growth factors and extracellular matrix affect the repair and regeneration process. Notably, studies involving the regulatory role of transcription factor TFAP2A indicating Schwann cell phenotypic changes post‐nerve injury have provided important insights [42]. Additionally, emerging studies are exploring the intricate interplay between the immune system and neuropathic pain, with the ultimate goal of identifying novel therapeutic strategies [43].

## 3 Pathophysiology of Cortical Plasticity in Peripheral Nervous Injury

### 3.1 Neural Pathways Affected by Peripheral Nervous Injury

An injury to the peripheral nervous system damages many neural pathways that considerably affect the central nervous system (CNS). Among the disrupted pathways, one of the most important pathways affected is the corticospinal tract which plays a major role in motor control. Patients diagnosed with leprosy, a disease predominantly affecting the skin and peripheral nerves, showed a change in handgrip muscle representation in the primary motor cortex (M1) [44]. According to this study, the most affected hand of these patients shows worse dynamometry performance. Furthermore, the motor threshold and motor‐evoked potential amplitude are modified. Findings suggest corticospinal pathway disruption.

Following peripheral nerve injury, significant alterations in sensory information processing within the somatosensory cortex can occur. For instance, in patients with traumatic median nerve injury, vibration‐induced neuropathy, or hand immobilization, studies have observed enlarged activation in the contralateral S1 and a loss of inhibition in the ipsilateral S1 during tactile stimulation [45]. The research suggests that both brain hemispheres respond to changes in afferent nerve signaling arising from the hand. This may be because the inhibition in the ipsilateral S1 is reduced, which plays an important role in brain adaptation.

Pain pathways are significantly affected, as demonstrated in a model of L5 spinal nerve ligation. Intrathecal administration of glial cell line‐derived neurotrophic factor (GDNF) successfully reversed mechanical allodynia, coinciding with a reduction in neuropeptide Y (NPY) upregulation within the touch‐sense pathway [46]. NPY enhances touch‐sense processing via Y1 receptor activation in the gracile nucleus following PNI, suggesting that GDNF's anti‐allodynic effects may be mediated through this mechanism.

In compressive injuries such as CTS, mechanical entrapment induces abrupt sensory loss, rapidly engaging four canonical plasticity paradigms: (1) unmasking of GABAergic suppression in S1, allowing latent horizontal connections to emerge within days; (2) representational expansion of adjacent cortical territories (e.g., digit remapping into spared nerve domains); (3) bilateral recruitment of contralateral homologous areas; and (4) misguided axonal sprouting from undamaged afferents into denervated zones [47]. This subtype's ‘burst‐like’ deafferentation accelerates synaptic remodeling (initial potentiation typically observable via electrophysiology within weeks), mediated by heightened neuronal excitability and microglial priming [36, 48]. Conversely, metabolic injuries (DPN/CIPN) feature protracted axonal demyelination and distal dying‐back, yielding slower, inflammation‐dominated plasticity (typically > 3 months for detectable S1 structural changes), prone to maladaptive outcomes such as ectopic hyperactivity and central sensitization via amplified N‐methyl‐D‐aspartate (NMDA) receptor trafficking [38, 49, 50].

### 3.2 Molecular and Cellular Mechanisms of Cortical Plasticity

At the molecular level, numerous genes and proteins are involved in cortical plasticity following PNI. Injury‐specific “switch” molecules and pathways further delineate these divergences, positioning BDNF as a pivotal regulator. In male mice, microglial BDNF mRNA expression in S1 is upregulated following peripheral nerve injury. The conditional knockout of BDNF in microglia has been shown to prevent injury‐induced synaptic remodeling [48]. Pyramidal neuron hyperactivity in the S1 cortex, along with pain hypersensitivity, has been attributed to BDNF signaling. In compressive models, BDNF upregulated by activated microglia within 1–4 weeks post‐injury serves as a pro‐plasticity switch, enhancing TrkB signaling to promote synaptic consolidation and dendritic arborization in S1 layer II/III neurons, thereby accelerating adaptive remapping [48].

By contrast, in metabolic injuries such as DPN and CIPN, chronic hyperglycemia or neurotoxic chemotherapeutic agents lead to progressive BDNF dysregulation and neurotrophic support failure, resulting in impaired neurotrophin signaling that promotes maladaptive S1 reorganization rather than adaptive plasticity [37, 49]. Whereas compressive subtypes exhibit rapid, BDNF‐mediated synaptic remodeling and S1 cortical map reorganization driven by microglial BDNF upregulation [47, 48], metabolic subtypes are characterized by chronically suppressed neurotrophin tone, delayed synaptic remodeling, and S1 gray matter reduction with aberrant somatotopic shifts [37]. A recent multilevel analysis of the central‐peripheral‐target organ pathway validated BDNF's pivotal role in post‐PNI recovery [47], while neurotrophin‐focused reviews in DPN and CIPN highlight the therapeutic potential of restoring Trk/p75NTR signaling balance [49]. These insights advocate for subtype‐targeted strategies: BDNF‐mimetic or microglia‐modulating approaches for compressive cases, and neurotrophin signaling restoration for metabolic cases, to optimize therapeutic windows (Table 1).

**TABLE 1:** Comparison of cortical plasticity mechanisms across peripheral nerve injury subtypes.

Injury subtype	Example	Synaptic remodeling speed	Key “Switch” molecule	Primary pathways	Supporting evidence
Compressive	CTS	Acute/subacute; rapid (2–8 weeks S1 reorganization)	BDNF (microglia‐upregulated, pro‐adaptive)	BDNF‐mediated synaptic remodeling and S1 cortical map reorganization	Flondell et al. (2024); Song et al. (2025); Huang et al. (2021) [36, 47, 48]
Metabolic	DPN/CIPN	Chronic, progressive (S1 gray matter reduction with maladaptive somatotopic reorganization)	BDNF (chronically dysregulated; neurotrophic support failure)	Impaired neurotrophin signaling promoting maladaptive S1 reorganization	Selvarajah et al. (2019); Samaddar et al. (2025) [37, 49]

It should be noted that certain molecular regulators of cortical plasticity have been well characterized in other cortical regions and may provide useful references for understanding S1/M1 plasticity after PNI. For example, the NRG1/ErbB4 signaling pathway in parvalbumin‐expressing inhibitory neurons has been shown to regulate critical period plasticity in the visual cortex [51], and experience‐dependent structural remodeling at both presynaptic and postsynaptic sites of layer 2/3 neurons has been demonstrated following visual deprivation [52]. Whether similar mechanisms involving inhibitory circuit remodeling and structural plasticity contribute to S1/M1 reorganization after PNI remains to be investigated.

In addition to microglial BDNF signaling, the cyclic GMP‐AMP synthase (cGAS)‐stimulator of interferon genes (STING) pathway also regulates macrophage activation and polarization [53]. cGAS‐STING detects cytosolic double‐stranded DNA released from damaged cells and acts as a double‐edged sword in sterile inflammation. Moderate activation supports physiological tissue remodeling, while chronic overactivation drives inflammation and fibrosis [54]. In the context of macrophage polarization, cGAS‐STING signaling promotes the pro‐inflammatory M1 phenotype, whereas suppression of this pathway favors the reparative M2 phenotype. M1‐polarized macrophages release TNF‐α and IL‐6, which engage the NF‐κB and JAK–STAT cascades [55]. These same cascades have been discussed above as contributors to maladaptive cortical plasticity after PNI. M2 macrophages resolve inflammation and support tissue remodeling [56]. In neuromuscular disorders, disruption of this M1/M2 balance leads to sustained inflammation and impaired functional recovery. It remains to be determined whether cGAS‐STING‐dependent macrophage polarization, through the cytokine‐NF‐κB axis described above, contributes to the transition from adaptive to maladaptive cortical plasticity after PNI.

Cellular stress responses also influence cortical plasticity after PNI. The AMPK/SIRT1/PGC‐1α pathway serves as a central regulator of cellular energy homeostasis and mitochondrial function [57]. Under metabolic stress, AMPK activation elevates NAD^+^ levels and activates SIRT1. SIRT1 then deacetylates PGC‐1α to drive mitochondrial biogenesis and oxidative metabolism. Disruption of this pathway has been linked to mitochondrial dysfunction and impaired synaptic plasticity in neurodegenerative conditions. Endoplasmic reticulum (ER) stress and the unfolded protein response (UPR) represent another stress signaling system relevant to neuronal function. ER stress and UPR activation contribute to skeletal muscle atrophy across a range of conditions, including disuse, denervation‐related neuromuscular diseases, and aging [58]. Whether similar ER stress mechanisms operate in central neurons following PNI, and whether AMPK/SIRT1/PGC‐1α activation or UPR modulation can restore adaptive cortical plasticity after PNI remains to be investigated.

### 3.3 Role of Neurotransmitters in Cortical Plasticity

Neurotransmitters play a vital role in modulating cortical plasticity. Dopamine, for instance, serves as a key regulator of motor cortex plasticity and motor skill learning. In the rat M1, both D1 and D2 receptor activities influence motor skill acquisition and long‐term synaptic potentiation, primarily through PLC activation. Importantly, the inhibition of PLC prevents the learning of a new forelimb reaching task, and coadministration of a PLC agonist can restore the learning deficits of the skill and dopamine antagonist‐induced reversal of synaptic plasticity [12].

ACh also plays a significant role in such processes. The insular cortex controls ACh release in taste learning and taste memory retrieval. When exposed to new taste stimuli, the release of ACh is greatly increased and GABAergic inhibition exerts strong control over cholinergic transmission. Remarkably, the patterns of cholinergic and GABAergic neuroactivity in the cortex following exposure to novel tastes are the opposite of those generated by familiar tastes [59]. The discovery of an inverse relationship between noradrenergic and dopaminergic systems shows that the roles of these neurotransmitter systems in memory are distinct.

Glutamate, the major excitatory neurotransmitter, plays a pivotal role in long‐term synaptic plasticity under both physiological and pathological conditions. In the anterior cingulate cortex (ACC) and insular cortex (IC), metabotropic glutamate receptor subtypes (mGluRs) are integral to synaptic modulation and plasticity associated with pain processing. Interestingly, mGluRs activation can induce LTD in chronic pain areas of the brain, making them a promising therapeutic target for chronic pain [60].

## 4 Diagnostic Techniques for Assessing Cortical Plasticity

### 4.1 Neuroimaging Modalities in Cortical Plasticity

Neuroimaging modalities have become indispensable tools for assessing cortical plasticity. fMRI is especially adept at detecting changes in brain activity related to plasticity. In a study conducted in individuals with upper‐limb amputation, fMRI was used to investigate changes in brain network functional connectivity [61]. The results showed changes in intra‐ and inter‐network functional connectivity, including decreases in intra‐network functional connectivity within selected regions and inter‐network connectivity between selected networks. These connectivity changes were associated with pain‐related clinical symptoms.

Diffusion tensor imaging (DTI) reflects the microstructural properties of white matter and can be used to study the structural changes associated with cortical plasticity. In a study of rats with peripheral nerve injury, DTI was used to investigate the diffusion plasticity of afferent and efferent pathways [62]. It indicated that electroacupuncture treatment in the injured rats changed the values of fractional anisotropy (FA) and radial diffusivity (RD) in the corticospinal tract and other regions such as the spinothalamic tract and internal capsule. These findings suggest an enhanced recovery of sensory and motor neural pathways, indicating that DTI may help to understand the processes of neural repair and plasticity.

Magnetic resonance spectroscopy (MRS) is a powerful method capable of measuring the concentration of certain brain metabolites that may be implicated in cortical plasticity. Although less commonly utilized compared to fMRI and DTI, MRS holds the potential to elucidate the biochemical changes associated with plasticity. For instance, in certain neurological conditions, alterations in metabolite levels detected via MRS may reflect underlying plasticity‐related changes in the brain, offering valuable insights into the mechanisms of neural adaptation and recovery [63].

### 4.2 Electrophysiological Methods for Evaluating Cortical Changes

Electrophysiology provides valuable insights into how the cortex changes functionally when undergoing plasticity. EEG is the recording of electrical activity from the scalp. EEG recordings in patients with spinal cord injury and neuropathic pain showed reduced peak frequency activity in the alpha band in frontal regions, indicating alterations in cortical activity [63].

Transcranial magnetic stimulation (TMS) is a commonly used noninvasive neurostimulation technique to study cortical excitability and plasticity. For instance, paired associative stimulation via TMS (TMS‐PAS) can induce LTP‐like plasticity. In a seminal study of schizophrenia, it was demonstrated how TMS‐PAS can induce LTP‐like plasticity. The neural mechanism behind cognitive deficits in schizophrenia was also investigated. Varenicline is a selective nicotinic ACh receptor partial agonist. It produces opposite effects on LTP in individuals with schizophrenia compared to healthy subjects [64]. These findings suggest its potential therapeutic utility in schizophrenia. These findings provide greater insights into the pathophysiology of schizophrenia and the development of novel therapeutic strategies.

Intracortical recordings enable researchers to characterize the functional properties of individual neurons, or small populations of neurons, in great detail. In preclinical research, it was shown that intracortical recordings are important to assess the impaired neuronal activity in the cortex after peripheral nerve injury. To analyze the spatiotemporal firing patterns of neurons after peripheral nerve injury, a study performed an intracortical recording and highlighted neuronal mechanisms underlying alterations in motor function. Thus, the study performed extensive recordings from the cortex [65]. The findings provide important insights into cortical reorganization and plasticity following peripheral neuropathies.

### 4.3 Biomarkers for Cortical Plasticity in Peripheral Nervous Injury

Biomarkers represent objective measures for assessing cortical plasticity in the context of peripheral nervous system injury. A notable biomarker in this regard is BDNF. Preclinical investigations have demonstrated that microglial‐derived BDNF in the S1 plays a critical role in mediating nerve injury‐induced synaptic remodeling, hyperactivity of pyramidal neurons, and pain hypersensitivity. The targeted removal of microglial BDNF effectively prevented the emergence of these pathological changes, underscoring the potential utility of BDNF as a biomarker in experimental models, though its clinical translation is impeded by variable blood‐CSF correlations, lack of assay standardization, and inconsistent sensitivity among different injury subtypes [48]. These findings highlight the importance of BDNF in the pathophysiology of chronic pain following peripheral nerve injury and its potential as a diagnostic and therapeutic target.

Despite promising preclinical data, translation of BDNF as a reliable biomarker faces several clinical hurdles. First, correlation between peripheral and CNS or CSF levels of BDNF is inconsistent across injury types and disease contexts: some studies in psychiatric or CNS‐disease cohorts show moderate positive correlation [66], while many studies in peripheral neuropathy are lacking such paired measurements. Second, preanalytical and analytical method heterogeneity—different blood matrices, choice of ELISA kits vs. ultrasensitive assays, recognition of mature vs. pro‐BDNF species—leads to large interstudy variability. For example, Polacchini et al. demonstrated that out of six assays tested, inter‐assay CV ranged 5%–20%, and only some kits distinguished mature BDNF specifically [67]. Third, confounding factors such as time of sampling, storage duration, platelet activation status, age, sex, and metabolic profile also modulate peripheral BDNF independent of neural injury. Therefore, specificity and sensitivity across injury subtypes and correlation with functional recovery remain under‐characterized.

Peripherin has also been identified as a potential biomarker in the context of peripheral nervous system injury. This protein is predominantly expressed in peripheral nerve axons, making it a highly specific marker for axonal integrity. In vitro models of axonal and demyelinating nerve injury have demonstrated that peripherin levels are significantly elevated only in cases of axonal damage, suggesting its specificity for detecting axonal injury. Interestingly, in patients with Guillain‐Barré syndrome, peak peripherin levels were significantly higher compared to other patient groups, further supporting its potential utility in acute axonal peripheral neuropathies. However, application to other neuropathy subtypes such as DPN or CIPN remains to be validated, and sensitivity in these contexts has yet to be established [68]. These findings highlight the importance of peripherin as a candidate biomarker for assessing axonal injury in both experimental and clinical settings. Future studies should focus on validating its diagnostic accuracy and exploring its role in monitoring therapeutic interventions.

While peripherin shows strong specificity for peripheral axonal damage in acute inflammatory neuropathies such as GBS [68], its performance in metabolic neuropathies (DPN), toxic neuropathies (CIPN), and chronic axonal degeneration remains untested. There is no published study reporting peripherin levels in serum/plasma of DPN or CIPN patients to establish sensitivity, specificity, or correlation with functional recovery. Thus, the generalizability of peripherin as a biomarker across injury subtypes is currently limited.

Markers related to neural activity are also valuable biomarkers for assessing cortical plasticity as well as neuropathic pain. For instance, preclinical studies exploring neuropathic pain due to PNI have shown significant changes in cortical surface morphometry and hierarchical topology [69]. This study found that cortical thinning in frontal and sensorimotor cortices and reduced gyrification in the insula correlate positively with the severity of neuropathic pain. The structural changes in these markers indicate that they may not only reflect changes in neural activity but also serve as sensitive indicators of cortical plasticity in the context of neuropathic pain due to peripheral nerve injury.

## 5 Therapeutic Strategies for Enhancing Cortical Plasticity

### 5.1 Pharmacological Interventions in Cortical Plasticity

**FIGURE 2:**
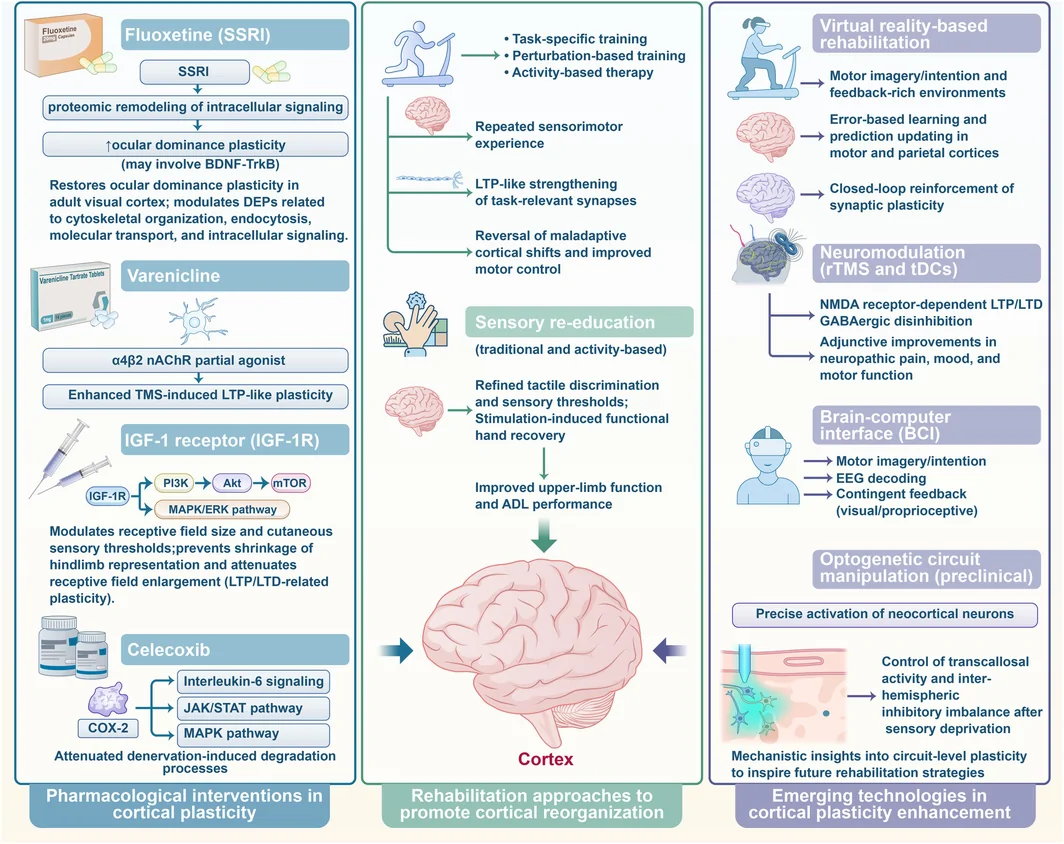
Therapeutic strategies and mechanistic pathways for enhancing cortical plasticity.

Pharmacological interventions are designed to modulate the molecular and cellular mechanisms that underpin cortical plasticity (Figure 2). Antidepressants represent a promising class of agents in this context. For example, the selective serotonin reuptake inhibitor fluoxetine restores ocular dominance plasticity in the adult mouse visual cortex. A broad proteomic analysis revealed the differentially expressed proteins (DEPs) with respect to controls that were associated with cytoskeletal organization, endocytosis, molecular transport, and intracellular signaling [70]. These findings suggest an impact on neuronal signaling pathways implicated in plasticity. Although the precise molecular cascade remains to be fully elucidated, these proteomic changes may involve BDNF‐mediated signaling, given the established role of BDNF–TrkB pathways in activity‐dependent cortical plasticity [8]. However, in human RCTs, evidence is mixed: a meta‐analysis of nine trials (*n* = 6788) shows that fluoxetine significantly improves motor function (Fugl‐Meyer Motor Scale) and functional independence (Barthel Index), but does not significantly reduce disability as measured by the modified Rankin Scale (mRS) at the end of treatment [71]. The evidence is graded as Low owing to indirectness (extrapolation from post‐stroke populations to PNI), heterogeneity in patient selection, dosage, and duration, and potential adverse effect risk [72].

An alternative therapy is the use of medication to manipulate neurotransmitters. Varenicline, a partial α4β2 nicotinic ACh receptor agonist, has been studied in nonsmoking patients with schizophrenia. In comparison to neurotypical controls, this compound modulated LTP‐like plasticity (measured with TMS paradigms) differently [64]. Importantly, administration of varenicline significantly boosted LTP‐like plasticity in the schizophrenia group, indicating its potential to alleviate the core cognitive deficits of the disorder. Although the crossover study in nonsmoking patients with schizophrenia demonstrated the modulation of LTP‐like plasticity, functional recovery outcomes were not assessed [64]. There is no RCT evidence in peripheral nerve injury or neuropathy settings; thus the GRADE evidence level for varenicline in these contexts remains Very Low, and its use should be considered experimental.

Pharmacological agents modulating neurotrophic signaling pathways represent a further avenue for augmenting cortical plasticity. For example, research investigating insulin‐like growth factor‐1 (IGF‐1) demonstrated that chronic intracortical IGF‐1 infusion in rats subjected to sensorimotor restriction modulated both receptive field dimensions and cutaneous sensory thresholds. IGF‐1 binding to its receptor (IGF‐1R) activates the PI3K/Akt/mTOR signaling cascade, a canonical pathway implicated in synaptic protein synthesis and plasticity [73]. Furthermore, this intervention prevented shrinkage of the hindlimb somatotopic representation and attenuated receptive field enlargement, indicating that IGF‐1 constitutes a fundamental modulator of cortical plasticity mechanisms [74].

Anti‐inflammatory strategies may also contribute to cortical plasticity enhancement. Celecoxib, a selective COX‐2 inhibitor, attenuates denervation‐induced muscle atrophy by suppressing inflammatory cytokine production, reducing oxidative stress, and preserving microcirculation [75]. These findings suggest that targeting post‐injury inflammation can protect neuromuscular function after nerve injury. The cytokine IL‐6 illustrates the complexity of immunomodulatory therapy. During acute exercise, IL‐6 acts as a beneficial myokine that supports muscle regeneration and metabolic adaptation. In chronic pathological states such as denervation and persistent inflammation, sustained IL‐6 elevation drives muscle atrophy via JAK/STAT and MAPK pathways [76]. This dual role indicates that the timing and context of cytokine modulation are critical for therapeutic application. Whether anti‐inflammatory agents or context‐dependent cytokine modulators can complement existing plasticity‐enhancing treatments such as fluoxetine or repetitive transcranial magnetic stimulation (rTMS) remains to be investigated.

### 5.2 Rehabilitation Approaches to Promote Cortical Reorganization

Neurorehabilitation strategies constitute a critical intervention for promoting experience‐dependent cortical reorganization following PNI. Targeted physical therapy modalities, including structured exercise regimens, demonstrably facilitate neuroplastic changes. For example, perturbation‐based balance training in Parkinson's disease patients significantly enhanced postural stability and reduced fall incidence. Furthermore, this training modulated both feedforward (anticipatory) and feedback (reactive) postural responses during stance and gait tasks, indicating facilitation of balance control via experience‐dependent cortical reorganization [77].

Occupational therapy interventions, incorporating sensory re‐education techniques, represent a further modality for augmenting cortical plasticity. Traditional sensory re‐education and activity‐based sensory re‐education constitute two principal modalities within this domain. These therapeutic strategies aim to enhance sensory discrimination and motor function in individuals with peripheral neuropathy through the facilitation of cortical reorganization. Supporting this, a scoping review of upper‐extremity peripheral nerve injury rehabilitation identified both modalities as being firmly grounded in empirical and theoretical frameworks for inducing cortical plasticity [78].

### 5.3 Emerging Technologies in Cortical Plasticity Enhancement

Novel technological platforms present innovative paradigms for modulating cortical plasticity. Virtual reality (VR) constitutes a prominent exemplar within this domain. A meta‐analysis confirmed significant therapeutic efficacy of VR modalities (including Wii Fit, ARMEO Spring, Rehabilitation Gaming System, and ArmAble) for augmenting functional capacity relative to control interventions [79]. For example, VR‐based neurorehabilitation programmes have been shown to enhance functional recovery in stroke survivors. Meta‐analyses involving 55 RCTs report moderate effect sizes for upper limb motor function and activities of daily living, with higher benefits when interventions are fully or semi‐immersive and when duration exceeds 6 weeks [80]. However, most of the included RCTs exhibit high heterogeneity and risks of performance/detection bias due to lack of patient blinding; follow‐up periods are usually short, limiting evidence on long‐term maintenance of gains [81].

Neuromodulatory techniques, including TMS and transcranial direct current stimulation (tDCS), represent emerging noninvasive therapeutic modalities. Notably, a recent dose–response meta‐analysis of randomized controlled trials (RCTs) demonstrated that rTMS over the left dorsolateral prefrontal cortex is superior to sham stimulation for treatment‐resistant depression (standardized mean difference 0.77, 95% CI 0.56–0.98), with efficacy moderated by stimulation frequency and total pulse dose [82]. In TBI populations, a systematic review and meta‐analysis of 12 RCTs reported moderate evidence for rTMS in reducing neuropathic pain and improving depressive symptoms [83]. For post‐stroke motor recovery, a meta‐analysis of RCTs found significant improvements in motor outcomes [84]. The overall evidence across these populations remains protocol‐dependent, yielding modest‐to‐moderate clinical effects (Table 2). Nevertheless, human RCTs specifically targeting peripheral nerve injury are scarce.

**TABLE 2:** GRADE‐based clinical evidence for therapies enhancing cortical plasticity.

Therapy	Clinical population	Evidence base	Overall effect on clinical outcomes	GRADE level	Supporting evidence
Fluoxetine	Post‐stroke recovery (subacute)	RCTs Meta‐analysis	Small‐moderate motor gains and improved functional independence (BI); no significant reduction in disability (mRS)	Low	Liu et al. (2021) [71]
Varenicline	Schizophrenia (nonsmokers; TMS plasticity paradigms)	1 randomized crossover trial	Increases LTP‐like cortical plasticity; functional recovery outcomes not tested	Very Low	Bridgman et al. (2016) [64]
VR‐based rehabilitation	Stroke motor rehabilitation	RCTs + Meta‐analyses	Improved upper limb motor function and functional independence compared with conventional therapy	Low‐Moderate	Soleimani et al. (2024); Peng et al. (2021) [80, 81]
rTMS	Stroke/TBI/Depression/Neuropathic pain	RCTs + Meta‐analyses	Modest‐moderate effects on pain, mood, and some motor outcomes; protocol‐dependent	Low‐Moderate	Yu et al. (2024); Li et al. (2022); Zhang et al. (2025) [82, 83, 84]
BCIs	Stroke and spinal cord injury	RCTs/Pilot studies	Promising motor gains; high variability and limited long‐term follow‐up data	Low	Sun et al. (2025); Ma et al. (2024); Brunner et al. (2024) [85, 86, 87]

Brain‐computer interfaces (BCIs) represent an emerging frontier for therapeutic neuromodulation. The DiSCIoser trial employs BCI‐supported motor imagery training to engage sensorimotor networks and facilitate neuroplasticity in cervical spinal cord injury patients [88]. A recent meta‐analysis of noninvasive BCI interventions in spinal cord injury (4 RCTs plus 5 self‐controlled trials, *n* = 109) reported significant improvements in motor function (SMD 0.72), sensory function (SMD 0.95), and activities of daily living (SMD 0.85), though the authors emphasize that these conclusions remain preliminary pending larger confirmatory RCTs [85]. In stroke populations, BCI interventions have yielded promising results in terms of sensorimotor cortical activation and motor outcome improvements, but currently exist only in small trials and pilot studies [86, 87]; thus the overall GRADE evidence for BCI therapies is Low. Functional recovery variability is high depending on lesion site, chronicity, patient engagement, and residual cortical capacity.

The integration of optogenetics has demonstrated remarkable potential in advancing our understanding of neural circuits. In a series of well‐designed experiments employing a rat model of sensory deprivation, optogenetic tools were successfully utilized to achieve precise activation of neocortical neurons, thereby enabling the systematic manipulation and regulation of transcallosal activity. This approach not only mitigated the adverse inhibitory effects observed in the deprived cortex but also revealed the critical role of transcallosal projections in maintaining interhemispheric communication [89]. These findings hold considerable promise for the development of novel and more effective rehabilitation strategies aimed at restoring cortical function in clinical settings.

### 5.4 Individualized Treatment Considerations and Evidence Assessment Framework

The selection of appropriate therapeutic strategies must be guided by patient‐specific factors including injury type, severity, timing, comorbidities, and potential risks of adverse events to ensure an individualized treatment approach. Translating interventions from preclinical models to clinical application further requires a critical evaluation of the underlying evidence. To this end, the Grading of Recommendations, Assessment, Development, and Evaluations (GRADE) framework offers a systematic method for assessing the quality of evidence [90]. In line with this approach, we have applied the GRADE criteria to evaluate the major interventions discussed in this section, with the resulting evidence profiles summarized in Table 2. Evidence levels (High, Moderate, Low, Very Low) were rated based on the following domains: risk of bias, inconsistency, indirectness, imprecision, and publication bias. Only RCTs in humans were considered for evidence grading; findings from animal or mechanistic studies are included as supplementary support but were not incorporated into formal evidence ratings.

## 6 Controversies and Challenges in Cortical Plasticity Research

### 6.1 Debates on the Extent of Cortical Plasticity

One of the primary debates in cortical plasticity research pertains to the degree to which the adult brain can reorganize. Several studies indicate that the adult brain exhibits notable neuroplasticity, even after substantial injury. For instance, in stroke patients, rehabilitation can result in substantial functional recovery, attributed to cortical plasticity mechanisms. In addition, mild peripheral nerve injuries such as early CTS have been shown to induce what may be characterized as excessive or maladaptive plasticity. For example, a meta‐analysis of structural/functional imaging in CTS patients revealed reduced inter‐digit cortical separation in S1, blurred somatotopy, and decreased resting‐state connectivity relative to healthy controls, correlating with sensory conduction delays and symptoms of increased excitability [91]. Moreover, prospective intervention increased S1 activation and yielded measurable improvements in sensory discrimination, consistent with recruitment of additional cortical representations in response to altered afferent input [36].

Conversely, other researchers contend that plasticity in the adult brain is constrained relative to the developing brain. Recent studies now suggest that the aged adult brain exhibits a diminished capacity for experience‐dependent plasticity, often termed “plasticity resistance” Animal data indicate that aged Schwann cells have a reduced capacity for dedifferentiation, diminished secretion of neurotrophic support, delayed clearance of myelin debris, and slower remyelination [92]. Aged murine models further show delayed macrophage response and prolonged inflammatory states after nerve injury [93]. In human patients, especially with chronic peripheral neuropathy, imaging studies report cortical thinning and reduced gyrification in sensorimotor and pain‐related areas, which correlate with the degree of nerve degeneration, suggesting that beyond a certain age or chronicity, the cortex becomes less plastic or responsive to standard therapeutic interventions [69].

In neurodegenerative disorders, most notably Alzheimer's disease, the decline in cognitive function is closely linked to a diminished capacity for synaptic plasticity. Several researchers have raised the question of whether it is feasible to restore significant levels of plasticity in such conditions. A recent investigation focusing on Alzheimer's disease patients revealed a marked reduction in frontal gamma activity, which coincided with a significant impairment in LTP‐like plasticity and a pronounced deterioration in cognitive performance. This association underscores the critical question of whether targeted interventions might effectively enhance synaptic plasticity and subsequently mitigate the progression of the disease [94].

The specific cellular mechanisms that underlie synaptic plasticity are also an important point of debate. Investigations are ongoing into the extent to which synaptic modifications, such as LTP and LTD, contribute to synaptic plasticity, given their association with different signaling mechanisms and the presence of spatial and temporal contextual influences. For example, the role of different neurotransmitter systems, as well as the receptors they activate, on critical period plasticity is not yet well understood in the visual cortex [51]. In severe or long‐gap nerve transections, animal models demonstrate early and subacute supraspinal changes—including alterations in volume, microstructure, metabolite profiles, and neuronal activity—yet many of these plasticity measures appear to reach a plateau, with limited further recovery in the absence of surgical repair or intensive rehabilitation [95]. Similarly, human studies reveal that motor cortical representation expands in early postoperative months but shows attenuated growth thereafter, despite ongoing therapy [96].

### 6.2 Ethical Considerations in Cortical Plasticity Studies

Cortical plasticity research raises essential ethical concerns when carried out on humans due to the invasive procedures or interventions that pose potential risks. One of the most crucial issues is the well‐being and the safety of the participants especially when advanced neurointerventions are used in a trial such as deep‐brain stimulation. Stem cell therapy has been recognized as having therapeutic benefit for spinal cord injuries [97]. It is important to note, however, that it has raised several ethical issues. Moreover, safety concerns include inflammatory response, immune rejection, and tumorigenic potential. Thus, the solution to this problem may be effective and efficient first and foremost, rigorous ethical review and stringent safety protocols.

The use of animal models is another ethical concern related to scientific research [98]. Animal research is critical for a full understanding of the basic mechanisms of cortical plasticity. However, one must ensure that the animals are kept to high welfare standards. Research needs to minimize pain, distress, and suffering in animals. Furthermore, alternative methods should be explored as stated by the respective authority. In studies utilizing animal models of peripheral neuropathy, experimental models should be designed to minimize distress.

In addition, data privacy and deficiencies in informed consent are other pertinent issues. In studies that assess cortical plasticity with neuroimaging or other techniques, the privacy of participants' data must be safeguarded [99]. Informed consent should be obtained only when participants are informed about the nature of the research, its purpose, risks, and benefits.

### 6.3 Limitations of Current Research Methodologies

The present approaches for studying cortical plasticity have limitations. One limitation is the lack of standardized protocols. There are different methods of induction and measurement of cortical plasticity used by different researchers across studies. In TMS studies, for example, the variability of stimulation parameters including frequency, intensity and duration has led to inconsistent findings [100].

Another limitation is the small sample sizes used in many studies. This limits the statistical power of studies, making generalizable conclusions difficult to draw. In some research on the impact of drugs on brain plasticity, the number of participants is relatively small, which may render the study underpowered to detect minor but nonetheless important treatment effects [64].

## 7 Future Directions in Cortical Plasticity and Peripheral Nervous Injury

### 7.1 Potential for Personalized Medicine in Cortical Plasticity

Personalized medicine can help with cortical plasticity in the future. According to recent studies of genetic polymorphisms, specific polymorphisms are important for cortical plasticity. For example, the Val66Met variant in the BDNF gene is a strong determinant of recovery after stroke. In particular, Met allele carriers are more dependent on subcortical plasticity mechanisms, whereas the ValVal individuals rely predominantly on intracortical plasticity mechanisms. The genetic characteristics provide a basis for rehabilitation strategies that are specifically adapted to certain genotypes. Thus, such strategies can increase efficacy and improve functional outcomes. Personalized medicine offers potential for furthering the precision and effectiveness of neurorehabilitation interventions through the incorporation of genetic information into clinical practice [34].

Machine learning is an important part of personalized medicine that uses new methods to improve the efficiency of treatment and patient outcomes. According to a recent study on machine learning investigating differences between subjects, theta burst stimulation was shown to vary on the basis of baseline neurophysiological parameters in the lower limb motor cortex [101]. The results reveal that machine learning may discover new predictive features such as motor evoked potential (MEP) amplitude in the case of intermittent theta burst stimulation and intracortical facilitation (ICF) in the case of continuous theta burst stimulation. These findings can play an important role in creating personalized treatment strategies, ultimately enhancing treatment accuracy for patients with neurological diseases.

Another critical aspect pertains to the use of biomarkers in guiding personalized treatment approaches. Biomarkers such as BDNF and peripherin play a crucial role in reflecting an individual's cortical plasticity status [48, 68]. By conducting biomarker assessments, clinicians can gain valuable insights into a patient's condition, enabling them to select the most appropriate treatment paradigm, whether it involves pharmacological interventions, rehabilitation strategies, or a combination of both. This approach not only enhances treatment specificity but also contributes to improved clinical outcomes in neurological and psychiatric disorders.

Extracellular vesicles (EVs) are membrane‐bound nanovesicles that transfer proteins, lipids, and nucleic acids between cells [102]. Their cargo reflects the physiological state of their cells of origin and can serve as a molecular signature of pathological processes. In the setting of PNI, circulating microRNAs and EV‐encapsulated cargo represent emerging biomarker classes that may complement traditional protein markers such as BDNF and peripherin. Combining these molecular signatures with the machine learning approaches described above could strengthen biomarker‐guided individualized treatment.

### 7.2 Innovations in Neurorehabilitation Techniques

Recent advancements in neurorehabilitation techniques represent a substantial evolution in the management and treatment of cortical plasticity associated with PNI. One particularly noteworthy area of progress lies in the development of increasingly sophisticated neuromodulation approaches [103]. Among these, Transcranial Pulse Stimulation (TPS), a recently emerged noninvasive brain stimulation modality, has garnered significant attention within the scientific community. While its therapeutic efficacy in addressing neuropsychiatric disorders remains under active investigation, preliminary clinical evidence demonstrates substantial potential in modulating symptomatology across patient populations. This necessitates future research efforts to focus on systematically optimizing TPS parameters, with the goal of enhancing its capacity to facilitate and augment cortical plasticity.

**FIGURE 3:**
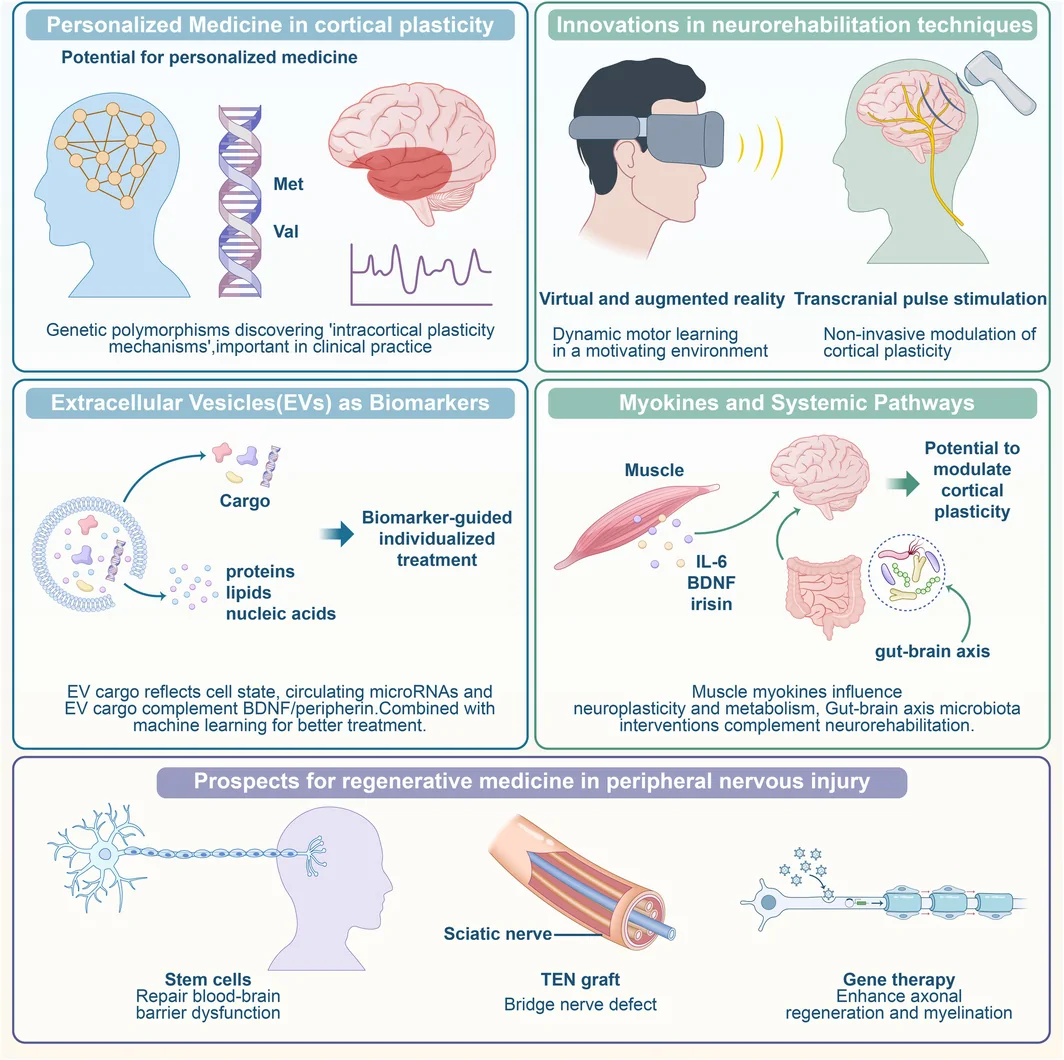
Future directions in plasticity and peripheral nervous injury.

Virtual reality (VR) and augmented reality (AR) are increasingly recognized as pivotal components in transforming neurorehabilitation paradigms. VR engages the patient in individualized, goal‐directed task practice within a motivating environment. VR creates an interactive, multisensory experience that immerses the user in the activity. For example, in Huntington's Disease rehabilitation, VR‐based cognitive training paradigms show considerable promise in improving cognitive outcomes. Moreover, the use of augmented reality technology supports the provision of feedback, guidance, and correction evolving over time, facilitating dynamic motor learning and enabling cerebral reorganization [104]. All these neurotechnological approaches have considerable potential to enhance modern rehabilitation techniques to optimize functional recovery and promote cortical plasticity and neuroregeneration (Figure 3).

The merging of different technology types is another growing trend for rehabilitation solutions. The emergence of brain‐computer interfaces (BCIs) with other complementary therapies, such as those used in physical therapy or pharmacotherapy, may yield better therapeutic outcomes. The BCI‐supported motor imagery training paradigm, which has been implemented in the DiSCIoser study as an add‐on to standard rehabilitation protocols, was found to enhance upper‐limb sensorimotor functional recovery of spinal cord injury patients [88]. These technologies may enhance the efficacy of rehabilitation and improve clinical outcomes.

Skeletal muscle functions as an endocrine organ that secretes myokines with effects on the brain. Myokines such as IL‐6, BDNF, and irisin participate in multi‐organ signaling networks that influence energy metabolism, neuroplasticity, gut microbiota composition, and immune function [105]. These muscle‐derived signals represent a pathway through which exercise and physical rehabilitation can promote cortical plasticity. Interorgan communication also involves the gut‐brain axis. Metabolic disturbances can alter gut microbiota composition and amplify systemic inflammation, as shown in studies of organ injury mediated by the gut‐lung axis in diabetes [106]. In the setting of PNI, where systemic inflammation and metabolic dysregulation are common, similar gut‐brain signaling mechanisms may affect rehabilitation outcomes. Exercise protocols, nutritional strategies, and microbiota‐targeted interventions may therefore complement existing neurorehabilitation approaches by engaging these systemic pathways.

### 7.3 Prospects for Regenerative Medicine in Peripheral Nervous Injury

Stem cells and regenerative medicine offer a novel way of treating peripheral nervous system injuries. For instance, umbilical cord‐derived mesenchymal stem cells (UC‐MSCs) show considerable potential for treating surgical wound‐related blood–brain barrier (BBB) dysfunction. Studies have suggested that transplanting UC‐MSCs can reduce the BBB permeability more effectively than IL‐6 antibody using a murine model in a preclinical study. In addition to their neuroprotective effects, UC‐MSCs have been shown to enhance wound healing [107]. The findings suggest that UC‐MSCs may serve as a novel and effective means of addressing PNI and its associated complications.

Another important area of focus in neuroregeneration is the creation of tissue‐engineered nerve grafts that provide a new solution to peripheral nerve injury. Researchers have engineered a novel bionic tissue‐engineered nerve (TEN) graft with biomimetic structural and compositional characteristics to mimic the remarkable properties of native nerves [98]. This new graft was able to repair damage in preclinical studies. It was transplanted in a rat to bridge a sciatic nerve defect. The outcome of the study revealed that TEN grafts have a reparative efficacy which is comparable to that of autografts.

Gene therapy also holds potential for peripheral nerve regeneration. A systematic review of animal studies found that BDNF‐based therapies, including viral vector‐mediated delivery, enhance axonal regeneration, myelination, and functional recovery after PNI [108]. Although most evidence remains preclinical, these approaches represent a direction for future regenerative treatment.

## 8 Conclusion

In conclusion, substantial progress has been made in understanding how peripheral nerve injury drives cortical reorganization. Compressive injuries rely on rapid BDNF‐mediated synaptic remodeling and S1 cortical map reorganization, while metabolic injuries involve chronically impaired neurotrophin signaling that drives maladaptive S1 reorganization. Neuroimaging and electrophysiological tools can now track these cortical changes over time, and emerging biomarkers such as BDNF, peripherin, and extracellular vesicle cargo provide additional means for patient evaluation. Systemic factors, including exercise‐induced myokines and gut microbiota composition, also affect cortical plasticity and can be incorporated into rehabilitation planning. Pharmacological agents, neuromodulation technologies, and structured rehabilitation protocols reveal subtype‐dependent effects, although their efficacy in PNI‐specific populations still requires confirmation through prospective trials. Further progress will depend on grouping patients by biomarker profiles, applying machine learning to multimodal datasets, and strengthening collaboration between basic research and clinical practice to develop individualized treatment for peripheral nerve injury.

## Author Contributions

C.C., D.G., and Y.S. contributed to the main structure of the manuscript; X.W., J.D., and Y.X. provided the references and drafted the manuscript; Z.J., Z.Y., and J.J. were responsible for auditing the manuscript. The authors have approved the final manuscript and agree to the authorship order.

## Funding

This work was supported by Natural Science Foundation of Jiangsu Province, BK20241158. Agricultural and Social Development Foundation of Hangzhou, 2024ZDSJ0136. Jiangsu Funding Program for Excellent Postdoctoral Talent, 2025ZB235.

## Conflicts of Interest

The authors declare no conflicts of interest.

## Data Availability

The data that support the findings of this study are available from the corresponding author upon reasonable request.
